# Estimation of central arterial pressure from the radial artery in patients undergoing invasive neuroradiological procedures

**DOI:** 10.1186/s12871-019-0844-1

**Published:** 2019-09-04

**Authors:** Sabino Scolletta, Antoine Herpain, Salvatore Mario Romano, Fabio Silvio Taccone, Katia Donadello, Boris Lubicz, Federico Franchi, Keitiane Michele Kaefer, Enrico Polati, Jean-Louis Vincent, Daniel De Backer

**Affiliations:** 1 Department of Intensive Care, Erasme Hospital, Université Libre de Bruxelles, Brussels, Belgium; 2 Department of Heart and Vessels, University of Florence, Azienda Ospedaliero-Universitaria Careggi, Florence, Italy; 3 Department of Interventional Neuroradiology, Erasme Hospital, Université Libre de Bruxelles, Brussels, Belgium; 4 Department of Medicine, Surgery and Neurosciences, Anesthesia and Intensive Care Unit, University Hospital of Siena, Siena, Italy; 5 Anesthesia and Intensive Care B, Department of Surgery, Dentistry, Paediatrics and Gynaecology, University of Verona, AOUI, University Hospital Integrated Trust of Verona, Verona, Italy; 6 Department of Intensive Care, CHIREC Hospitals, Université Libre de Bruxelles, Brussels, Belgium

**Keywords:** Central pressure waveform, Peripheral artery waveform, Arterial pressure, Pulse contour analysis

## Abstract

**Backgrounds:**

Central arterial pressure can be derived from analysis of the peripheral artery waveform. The aim of this study was to compare central arterial pressures measured from an intra-aortic catheter with peripheral radial arterial pressures and with central arterial pressures estimated from the peripheral pressure wave using a pressure recording analytical method (PRAM).

**Methods:**

We studied 21 patients undergoing digital subtraction cerebral angiography under local or general anesthesia and equipped with a radial arterial catheter. A second catheter was placed in the ascending aorta for central pressure wave acquisition. Central (AO) and peripheral (RA) arterial waveforms were recorded simultaneously by PRAM for 90–180 s. During an off-line analysis, AO pressures were reconstructed (AO_rec_) from the RA trace using a mathematical model obtained by multi-linear regression analysis. The AO_rec_ values obtained by PRAM were compared with the true central pressure value obtained from the catheter placed in the ascending aorta.

**Results:**

Systolic, diastolic and mean pressures ranged from 79 to 180 mmHg, 47 to 102 mmHg, and 58 to 128 mmHg, respectively, for AO, and 83 to 174 mmHg, 47 to 107 mmHg, and 60 to 129 mmHg, respectively, for RA. The correlation coefficients between AO and RA were 0.86 (p < 0.01), 0.83 (p < 0.01) and 0.86 (p < 0.01) for systolic, diastolic and mean pressures, respectively, and the mean differences − 0.3 mmHg, 2.4 mmHg and 1.5 mmHg. The correlation coefficients between AO and AO_rec_ were 0.92 (p < 0.001), 0.87 (p < 0.001) and 0.92 (p < 0.001), for systolic, diastolic and mean pressures, respectively, and the mean differences 0.01 mmHg, 1.8 mmHg and 1.2 mmHg.

**Conclusions:**

PRAM can provide reliable estimates of central arterial pressure.

**Electronic supplementary material:**

The online version of this article (10.1186/s12871-019-0844-1) contains supplementary material, which is available to authorized users.

## Introduction

Reliable arterial pressure monitoring is essential in critically ill patients. Physicians use the mean arterial pressure value as an index of tissue perfusion [1], but interpretation of arterial pressure waveform or derived variables is not always straightforward. In particular, the arterial pressure recorded from a femoral or radial indwelling catheter differs somewhat from the central (i.e., aortic) pressure, which is a key determinant of left ventricular afterload and coronary perfusion [2, 3]. Reliable assessment of central arterial pressure has been a topic of recent investigation; in particular, aortic pressure is better related to the severity of atherosclerosis, loading conditions of the left ventricular myocardium, and left ventricular and vascular remodeling than are conventional peripheral pressures. It is also a better predictor of cardiovascular events and mortality in non-critically ill patients than peripheral pressure [4–6].

Critically ill patients experience significant variability in arterial pressure and changes in pressure wave morphology as a result of variations in arterial tone (e.g., from sepsis-induced vasodilation or post-surgical bleeding and vasoconstriction) [7] so that peripheral arterial pressure cannot be reliably correlated with absolute values of central arterial pressure [8]. In particular, increasing doses of vasoactive drugs may have different effects on central and peripheral arterial pressures [8–11].

Central arterial pressure can be assessed non-invasively by applanation tonometry, using a mathematical transformation (i.e., “transfer function”) to estimate the aortic pressure wave from the peripheral (i.e., brachial or radial) pulse wave [2, 12–15]. In this approach, brachial arterial pressure measurement is needed to calibrate the transfer function, and aortic blood pressure is derived taking into account the timing of both the antegrade and retrograde pulse waves [13, 14]. An alternative method is the Pressure Recording Analytical Method (PRAM), a pulse contour analysis system that can reconstruct the central arterial pressure using a mathematical model applied to the peripheral pressure wave [16]. Romano et al. showed that the function linking the central to the peripheral waveform and other variables (e.g., dicrotic pressure, diastolic pressure, heart rate, …) was sufficient to be able to estimate the central arterial pressure from the pressure recorded at the radial site [17], avoiding the need to reconstruct every individual arterial wave point. Thus, PRAM could be helpful in ICU patients who are only monitored with an indwelling peripheral arterial catheter as central arterial pressures could be estimated using a continuous beat-to-beat approach.

The aim of this study was therefore to compare actual measured central arterial pressures (from an intra-aortic catheter), peripheral arterial pressures (from a radial catheter) and central arterial pressures estimated by PRAM in patients undergoing invasive neuroradiology procedures.

## Methods

### Patients

The Erasme University Hospital Ethics Committee approved the study (number P2011/077) and written informed consent was obtained from all patients. We prospectively studied a convenience sample of 21 adult critically ill neurological patients equipped with a 20-gauge radial arterial catheter for arterial pressure monitoring and who required digital subtraction cerebral angiography for their neurological assessment and management. We excluded patients with pathologies that could affect the quality and reliability of the arterial signal, such as aortic valve pathologies, aortic aneurysms, and cardiac arrhythmias. We also excluded patients with poor quality arterial pressure signals as a result of excessive over- or underdamping of the catheter-transducer system, checked using the fast flush test (see next section for details) [18, 19].

### Study protocol

All angiograms were performed by the same neuroradiologist (B.L.) under local or general (using propofol infusion pumps) anesthesia, depending on the patient’s condition. In all patients, the 20G peripheral arterial line was placed on the day of the procedure. Femoral arterial access was used for introduction of the aortic catheter in each patient, with a 7F introducer followed by a 5F guiding catheter placed in the ascending aorta with contrast material injected to evaluate the location of the catheter tip. After confirmation of the correct position, the catheter guide was connected to a transducer system (Edwards Lifesciences, Irvine, USA) for arterial pressure wave acquisition. After zeroing the pressure transducer, the frequency response of the arterial blood pressure transducer was checked using a fast flush test. The test consists of a brief opening of the catheter-transducer system (fast flash valve) to the high-pressure (300 mmHg) saline bag, to obtain a transient square wave in the arterial signal [18]. Closure of the fast flush valve results in pressure oscillations, allowing computation of the natural frequency and damping coefficient [19, 20].

**Fig. 1:**
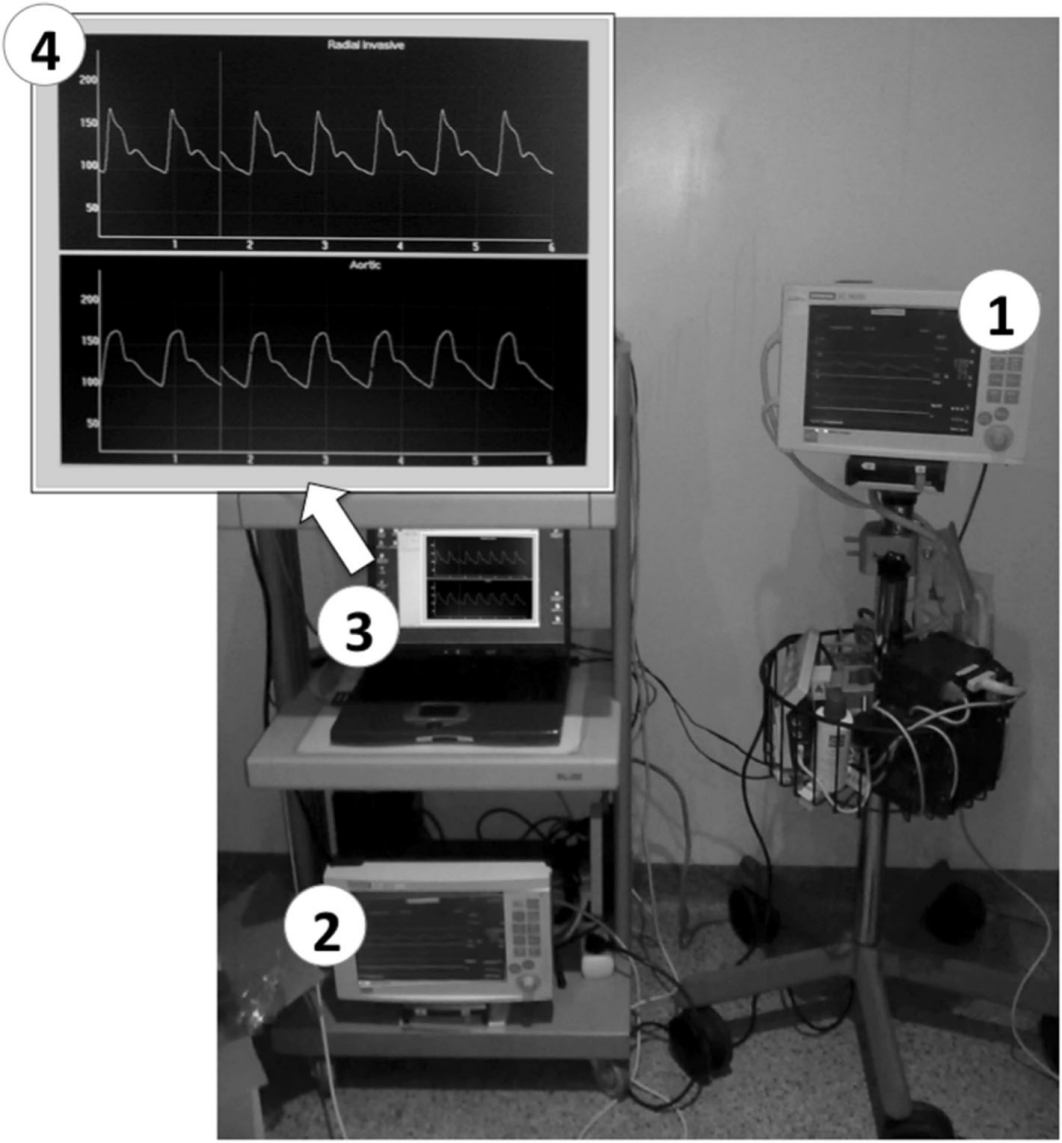
The central and the peripheral arterial waveforms were displayed on two main monitors (Siemens, SC 9000) (1 = central, 2 = peripheral). PRAM software was installed on a laptop (3) that was connected to both Siemens monitors to simultaneously record the aortic and radial pressure waves for 90–180 s. The PRAM screen (4) shows the simultaneous recordings of radial (top) and aortic (bottom) pressure waves in one of the patients enrolled in the study

The central and the peripheral arterial waveforms were displayed on two different monitors (Siemens, SC 9000) (Fig. 1). The PRAM system was connected to both monitors and the standard angiographic procedure was temporarily put on stand-by. All measurements were obtained during periods of hemodynamic stability (mean arterial pressure and heart rate variations < 5%). During this period, no changes in therapy or mechanical ventilation settings were allowed. The aortic and radial pressure waves were recorded simultaneously for 90–180 s to allow storage of a sufficient number of arterial pressure waves to construct the central arterial pressure values from the peripheral ones. At the end of the pressure wave recordings, the standard angiographic procedure was resumed and the PRAM disconnected.

### Pressure measurements

During an off-line analysis, aortic systolic pressure (SAP_ao_), aortic diastolic pressure (DAP_ao_), and aortic dicrotic pressure (Dic_ao_) were obtained directly from the measured aortic pressure wave. Peripheral arterial pressure values were obtained from the radial pressure wave (SAP_peripheral_, DAP_peripheral_, Dic_peripheral_, MAP_peripheral_, and PP_peripheral_). Because measurements were obtained during periods of hemodynamic stability, the average values of all aortic and peripheral pressure waves were calculated. Blood pressure values affected by extrasystolic heart beats (if any) were excluded from the averages. Digital aortic mean pressure (MAP_ao_) and pulse pressure (PP_ao_) measurements were calculated using standard formulas [MAP_ao_ = (SAP_ao_ + 2*DAP_ao_)/3 DAP_ao_ + PP_ao_/3; PP_ao_ = SAP_ao_-DAP_ao_].

Aortic pressure was also reconstructed from the radial arterial trace obtained by PRAM using a mathematical model obtained from a multi-linear regression analysis (see next section). The physician who performed the off-line analyses (SMR) was not aware of the reference aortic pressure measurements.

### Mathematical model for reconstructing the aortic pressure wave

For all the arterial pressures recorded, the off-line analyses were conducted using the PRAM software, assessing the frequencies and resonance points of the waveform morphology for the whole cardiac cycle. With PRAM, the arterial pressure signal is acquired at 1000 points per second (P/t, mmHg x sec^− 1^) and the aortic impedance (Z) is obtained from the morphologic analysis of both the pulsatile and continuous components of the pressure waveform. According to PRAM, Z(t) is equal to (P/t) x K(t) where P/t (mmHg x sec^− 1^) is a description of the pressure wave profile (the morphology) expressed as the variations in pressure (P) over time (t) and K is a factor inversely related to the instantaneous acceleration of the vessel cross sectional area (sec^2^ x cm^− 1^) x (1 x cm^− 2^). This factor represents the non-linear relationship between changes in the vessel P/t (mmHg x sec^− 1^) and the arterial pressure. K is obtained from the ratio between the expected (e.g., 100 mmHg at the periphery and 90 mmHg in the aorta) and the calculated mean arterial pressures [17].

Using data obtained during left heart catheterization in cardiac patients, Romano et al. previously showed that each point of the pressure waveform could be used to reconstruct the central aortic pressure using a multiple linear regression model to generate a transfer function from the periphery to the aorta [16, 17]. The multi-linear regression analysis was performed on a dataset of 123 measurements from 41 cardiac patients undergoing heart catheterization [16]. Peripheral and central pressure waves were recorded simultaneously in each patient (three pulsations each separated by 30 s). To work in the time domain, the single pulsations from peripheral and central records were interpolated linearly. The two signals were put in phase and the pressure and pulsation waveforms were analyzed. From each beat, samples were interpolated on a 1024 point grid. For each point a linear multiple regression was computed to obtain the single aortic waveform from the peripheral one [16]. For each variable (SAP_rec_, DAP_rec_ and Dic_rec_), the same formula was used: P_ao_ = a_0_ ± a_1_ · b_1_ ± a_2_ · b_2_ ± a_3_ · b_3_ ± …., where a_i (i = n-1)_ are constant, and b_j (j = n)_ are variables. For each point of the recorded peripheral pulse waveform, the reported multiple linear regression was applied. Independent variables were the radial pressure at the corresponding point, its first and second derivative, each point integral of radial systolic, diastolic, dicrotic and pulse pressure, heart rate, cardiac cycle efficiency [21] and Z (t1_instability_) [16, 17].

### Statistical analysis

Data are presented as mean and standard deviation (SD), median [IQRs] or count, as appropriate. Statistical analysis was performed using the software GraphPad Prism version 5.0 (San Diego, CA, USA). For continuous data, the normal distribution was evaluated using the Kolmogorov-Smirnov test before a Student’s paired t-test was used. The relationship between measured aortic and peripheral pressures was assessed using linear correlation analysis, and mean differences with standard errors (SE) and 95% confidence interval (CI) were calculated. Because the reference method was the measurement of the true pressure in the aorta rather than a method with an intrinsic error [22], a linear regression analysis could be used to test the relationship between reconstructed and measured aortic pressure. The regression coefficient (R^2^), 95% CI, and equation of regression were derived. In addition, in order to estimate the differences between measured and reconstructed arterial pressure values, mean bias, SE, and 95% CI were calculated. Statistical significance was considered as a p value of < 0.05.

## Results

A total of 25 acquisitions were obtained in 21 patients (Table 1). Three patients had repeated angiography on different days (two cases 1 day later and one case 8 days later) after embolization of a cerebral aneurysm. Individual values of central aortic pressures, peripheral pressures and reconstructed aortic pressures are given in the Additional file 1: Table S1. For all the acquisitions, the frequency response of the arterial blood pressure transducer was adequate using the fast flush test, so no arterial signals were excluded from the analysis.

**Table 1:** Demographic data, main diagnosis, comorbid diseases and severity scores on intensive care unit admission

Anthropometric data	
Age (years)	49 ± 15
Height (cm)	170 ± 9
Weight (Kg)	81 ± 21
Sex (M/F)	8/13
APACHE II Score	11 [3–25]
SOFA Score	3 [0–10]
Main diagnosis	
Subarachnoid hemorrhage	7
Unruptured cerebral aneurysm	13
Arterial-venous malformation	1
Comorbid diseases	
Arterial hypertension	11
Obesity	4
COPD	1

Data are presented as mean ± SD, median [IQRs] or count

*APACHE II Score* Acute Physiology and Chronic Health Evaluation II, *SOFA* Sequential Organ Failure Assessment, *COPD* Chronic obstructive pulmonary disease

SAP_ao_, DAP_ao_ and MAP_ao_ values ranged from 79 to 180 mmHg, 47 to 102 mmHg, and 58 to 128 mmHg, respectively. SAP_peripheral_, DAP_peripheral_, and MAP_peripheral_ values ranged from 83 to 174 mmHg, 47 to 107 mmHg and 60 to 129 mmHg, respectively. The correlation coefficients between SAP_ao_-SAP_peripheral,_ DAP_ao_-DAP_peripheral_, and MAP_ao_-MAP_peripheral_ were 0.86 (p < 0.01), 0.83 (p < 0.01) and 0.86 (p < 0.01), respectively (Additional file 1: Fig. S1). The differences between pressure values recorded in the aorta and the radial artery were − 0.3 mmHg (SE 2.5 mmHg), 2.4 mmHg (SE 1.6 mmHg), and 1.5 mmHg (SE 1.7 mmHg), for systolic, diastolic and mean pressures, respectively (Additional file 1: Table S2). In eight patients, the difference between SAP_ao_ and SAP_peripheral_ was > 10 mmHg; in 4 of these patients SAP_ao_ was > SAP_peripheral_. In 7 measurements (made in 6 patients) the difference between DAP_ao_ and DAP_peripheral_ was > 10 mmHg; in six of these measurements DAP_ao_ was > DAP_peripheral_.

**Fig. 2:**
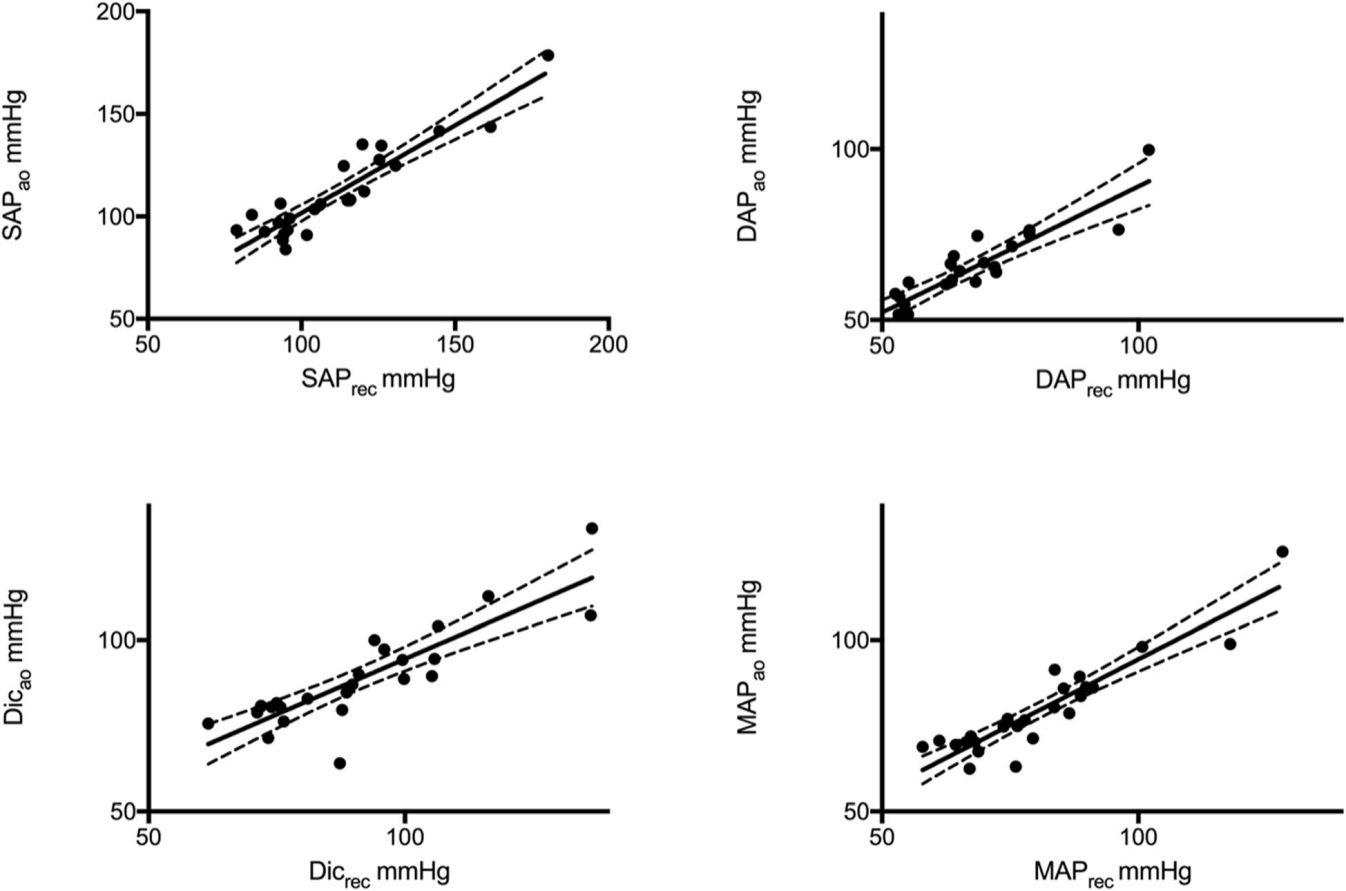
Linear regression of reconstructed (X-axis) and measured (Y-axis) central pressure values. Upper panels: left ➔ correlation between systolic pressure obtained from aorta (SAP_ao_) and reconstructed from peripheral artery (SAP_rec_); right ➔ correlation between diastolic pressure obtained from aorta (DAP_ao_) and reconstructed from peripheral artery (DAP_rec_). Lower panels: left ➔ correlation between dicrotic pressure obtained from aorta (Dic_ao_) and reconstructed from peripheral artery (Dic_rec_); right ➔ correlation between mean pressure obtained from aorta (MAP_ao_) and reconstructed from peripheral artery (MAP_rec_). The continuous lines show the straight correlation; dotted lines represent the 95% confidence intervals

The mean values of the reconstructed pressures (SAP_rec_, DAP_rec_, and MAP_rec_) are given in Table 2. There was excellent correlation between systolic, diastolic and mean arterial pressure values measured in the aorta and reconstructed from the radial artery (r = 0.92, < 0.001; r = 0.87, p < 0.001; r = 0.92, p < 0.001 respectively). Linear regression analysis between measured and reconstructed pressures is shown in Fig. 2 and in the Additional file 1: Table S3. The mean differences between SAP_ao_ and SAP_rec,_ DAP_ao_ and DAP_rec_, and MAP_ao_ and MAP_rec_ were 0.01 mmHg (SE 1.8), 1.8 mmHg (SE 1.3) and 1.2 mmHg (SE 1.3), respectively (Table 2). SAP_rec_ overestimated SAP_ao_ by > 10 mmHg in 5 patients and underestimated it by > 10 mmHg in 3 patients. DAP_rec_ underestimated DAP_ao_ by > 10 mmHg in 2 patients. MAP_rec_ overestimated MAP_ao_ by > 10 mmHg in 1 patient and underestimated it by > 10 mmHg in 2 patients (Additional file 1: Table S4).

**Table 2:** Mean arterial aortic pressure values recorded directly in the aorta and reconstructed from peripheral arterial waveforms

Variables	Aortic	Reconstructed	*P*	Bias	SE	95% CI
SAP mmHg	111.7 ± 24.1	111.7 ± 22.3	0.99	0.01	1.8	−3.8 to 3.8
DAP mmHg	65.5 ± 13.5	63.7 ± 11.5	0.18	1.8	1.3	−0.9 to 4.6
Dic_,_ mmHg	90.9 ± 19.4	88.6 ± 14.6	0.21	2.3	2.0	−1.8 to 6.5
MAP mmHg	80.9 ± 16.6	79.7 ± 13.9	0.37	1.2	1.3	−1.5 to 4.0
PP mmHg	46.5 ± 13.6	48.9 ± 16.2	0.13	−1.8	1.5	−5.0 to 1.4

Data are expressed as mean ± standard deviation, or mean, standard error (SE) and 95% confidence interval (CI)

*SAP* Systolic arterial pressure, *DAP* Diastolic arterial pressure, *Dic* Dicrotic pressure, *MAP* Mean arterial pressure, *PP* Pulse pressure

Subgroup analyses of the central aortic, peripheral and reconstructed aortic pressures according to norepinephrine therapy, history of arterial hypertension, subarachnoid hemorrhage on admission, and sex are given in Additional file 1: Tables S5-S8.

## Discussion

In stable patients undergoing an invasive neuroradiology procedure, there was a highly significant correlation between systolic, diastolic and mean arterial pressure values measured in the aorta and in the radial (peripheral) artery. There was also a significant correlation between measured values and reconstructed values obtained by PRAM using a specific mathematical model applied to the peripheral pressure wave. These data are novel since no studies have compared reconstructed aortic pressure values obtained using PRAM with directly measured values in this patient population.

Several studies have reported significant differences between central and peripheral arterial pressures, suggesting that peripheral pressure is a poor surrogate for central pressure [2, 8, 12–14]. Our results showed a good correlation between central and peripheral pressures, with a low bias between central and peripheral systolic and diastolic pressures. However, there was a greater than 10 mmHg difference between central and peripheral systolic pressures in about 30% of the measurements (40% of the patients). In critically and non-critically ill patients, the differences between central and peripheral systolic pressures range between 7 and 20 mmHg [23–25]. It has been shown in non-critically ill patients that the greater the ratio between systolic central and peripheral pressure, the poorer the outcome. Moreover, several factors have been reported to be associated with increased ratios, including comorbid diabetes, hypertension and cardiovascular disease [9, 26–29], although conflicting results exist [30]. In our sample of stable patients, we could not determine whether the bias was related to the severity of the patients’ acute condition, to an underlying chronic vascular disease or to a combination of both. In addition, the degree and type (i.e., local, general, inhalational, intravenous) of analgesia and anesthesia may have impacted differently on the central and peripheral arterial vessels, decreasing the peripheral vascular tone by different amounts and causing varying degrees of vasodilation. This would explain why some patients had a radial systolic blood pressure that was lower than their central pressure, although these differences were not statistically significant. Unfortunately, our patients were not equipped with neuromonitoring systems to assess the depth of the anesthesia.

We have shown that central pressure values reconstructed from peripheral pressure waveforms recorded and analyzed using PRAM correlated well with the directly measured ascending aorta pressure. The central pressure waveform is generally obtained using applanation tonometry. Our results are similar to those from studies comparing central pressure derived from applanation tonometry with direct aortic pressure measurements. As an example, Chen et al. applied a transfer function to reconstruct central pressure in 20 patients undergoing cardiac catheterization and reported a correlation coefficient (R) of 0.97 and a bias of 0.0 ± 3.7 mmHg [12]. In a similar population (n = 14), Karamanoglu et al. found a significant relationship (R^2^ = 0.95) and a difference of 2.4 mmHg (SE = 1 mmHg) between measured and reconstructed aortic pressure [23]. A recent meta-analysis analyzed the results of 22 studies, including a total of 857 patients, which compared applanation tonometry-estimated and directly measured central aortic pressure [31]. The mean difference between measured and reconstructed systolic arterial pressure was − 1.1 ± 4.1 mmHg (95% limits of agreement from − 9.1 to 6.9 mmHg) using an invasive calibration (i.e., obtained using mean and diastolic aortic pressure values). However, the mean differences increased to − 8 ± 10 mmHg when the pressure waveform was calibrated non-invasively using a sphygmomanometer.

Systolic, diastolic and dicrotic pressures analyzed by PRAM from the radial artery have previously been reported to accurately estimate aortic pressures in patients undergoing cardiac catheterization [16]. The authors also reported that the true aortic pressure wave and the reconstructed one had similar shapes. In the present study, we were not able to clearly demonstrate that the reconstructed pressures obtained by PRAM were closer to central pressures than were the peripheral measurements. In fact, we found similar correlations and biases between central and reconstructed, and central and peripheral pressures.

Our study has some limitations. First, because of our small sample size, we could only perform limited subgroup analyses and cannot determine whether differences in the reconstructed pressure waves could have been associated with various clinical factors (e.g., age, cardiac disease, history of hypertension, sedative agents, tachycardia, etc.…). Second, although the same fluid-filled systems were used to measure central aortic and peripheral pressures, the lengths and widths of the catheters inserted into the central and peripheral sites were different. Thus, artifacts as a result of an inappropriate dynamic response (harmonics, damping, etc) of the catheter-transducer systems may have affected the measurements. It has been shown that increasing the internal radius of the catheter decreases the damping coefficient [32]. Romagnoli et al. showed that the arterial catheter diameter (20- versus 18 gauge) was one of the parameters independently associated with underdamping/resonance; the smaller the diameter of the catheter, the greater the damping coefficient of the catheter-transducer system [32]. We are unable to state whether our results would have been the same using identical catheters and fluid-filled systems to measure the aortic and radial pressures. Pauca et al. previously used similar catheters (20G) to record the pressure waveforms in both the aorta and the radial artery in patients undergoing cardiac surgery. They reported similar mean differences to those recorded in the present study, for mean and diastolic pressures but not for systolic pressure, which resulted in higher values at the peripheral site; this is in agreement with basic hemodynamic principles [24]. However, by contrast with our study, Pauca et al. enrolled cardiac surgical patients undergoing standardized general anesthesia and analgesia [24]. Unfortunately, we did not record the natural frequency and damping coefficient obtained using the fast flush test. In addition, we did not use a high fidelity catheter [32]. Chen et al. demonstrated agreement and bias between measured and reconstructed aortic pressure using a micromanometer to measure aortic pressure similar to our study [12]; however, in the majority of studies, radial pressure was measured using a fluid-filled catheter. Third, we used the mean arterial pressure calculated from the standard formula instead of the value computed from the average of the arterial waveform. We chose this method because standard monitors used in clinical practice calculate the mean arterial pressure from systolic and diastolic pressures. Fourth, we investigated a population of stable patients undergoing the same procedure and the agreement between measured and reconstructed arterial pressure may differ more substantially in less stable patients. For example, in hypertensive, hypotensive and septic patients and in patients treated with vasoactive agents, changes in arterial tone may have different effects on the characteristics of arterial wall and waveform morphology due to alteration in reflected waves [7, 33–35]. Finally, we reconstructed central aortic pressure only from radial traces. Arterial pressure can have different values and waveforms at different sites [2], and we cannot be sure that applying PRAM on femoral arterial signals would give similar results.

In conclusion, in the present study performed in stable patients during cerebral angiography, central arterial pressure values reconstructed from the radial artery pressure wave using PRAM were similar to those measured directly in the aorta; however, given the small differences between measured peripheral and measured aortic pressures, we were unable to demonstrate conclusively that the PRAM technique could reconstruct the central arterial pressure. Further studies are warranted in patients with larger differences between measured peripheral and aortic pressures (e.g., vascular surgical or diabetes patients) to confirm validity. In addition, studies should also assess whether this method could be helpful in critically ill patients with different conditions that may affect the pressure wave shape (e.g., sepsis, cardiac failure, trauma, hemorrhage) and may increase the risk of bias between measured and estimated pressures.

## Additional file

Additional file 1: **Table S1.** Individual values of central aortic pressure, peripheral pressure and aortic reconstructed pressure. **Figure S1.** Correlations between systolic (SAP_ao_), diastolic (DAP_ao_) and mean (MAP_ao_) arterial pressure recorded in the aorta and those recorded in the radial artery (SAP_peripheral_, DAP_peripheral_, MAP_peripheral_). Dotted lines represented the 95% confidence intervals. **Table S2.** Differences between arterial pressures recorded in the aorta and those recorded in the radial artery. **Table S3.** Linear regression of measured and reconstructed central arterial pressure values. **Table S4.** Numbers of patients with > 10 mmHg differences between central and reconstructed pressures. **Table S5.** Arterial blood pressure according to norepinephrine therapy. **Table S6.** Arterial blood pressure according to the presence of chronic hypertension, defined as hypertension in antihypertensive therapy. **Table S7.** Arterial blood pressure according to the presence of subarachnoid hemorrage (SAH) on admission. **Table S8.** Arterial blood pressure according to patient sex. (DOCX 133 kb)

## Data Availability

The datasets used and/or analysed during the current study are available from the corresponding author on reasonable request.

## Abbreviations

AO: Central arterial waveforms
DAP_ao_: Aortic diastolic pressure
DAP_peripheral_: Radial diastolic pressure
DAP_rec_: Reconstructed diastolic pressure
Dic_ao_: Aortic dicrotic pressure
Dic_peripheral_: Radial dicrotic pressure
ICU: Intensive care unit
MAP_ao_: Aortic mean pressure
MAP_peripheral_: Radial mean pressure
MAP_rec_: Reconstructed mean pressure
PP_ao_: Aortic pulse pressure
PP_peripheral_: Radial pulse pressure
PRAM: Pressure Recording Analytical Method
RA: Peripheral arterial waveforms
SAP_ao_: Aortic systolic pressure
SAP_peripheral_: Radial systolic pressure
SAP_rec_: Reconstructed systolic pressure
Z: Aortic impedance
