# Supplementary material for: Estimation of central arterial pressure from the radial artery in patients undergoing invasive neuroradiological procedures
Source: BMC Anesthesiol. 2019 Sep 4;19:173. doi: 10.1186/s12871-019-0844-1 (PMC6727486; doi:10.1186/s12871-019-0844-1)
Supplement: Supplementary file 1 — Table S1. Individual values of central aortic pressure, peripheral pressure and aortic reconstructed pressure. Figure S1. Correlations between systolic (SAPao), diastolic (DAPao) and mean (MAPao) arterial pressure recorded in the aorta and those recorded in the radial artery (SAPperipheral, DAPperipheral, MAPperipheral). Dotted lines represented the 95% confidence intervals. Table S2. Differences between arterial pressures recorded in the aorta and those recorded in the radial artery. Table S3. Linear regression of measured and reconstructed central arterial pressure values. Table S4. Numbers of patients with > 10 mmHg differences between central and reconstructed pressures. Table S5. Arterial blood pressure according to norepinephrine therapy. Table S6. Arterial blood pressure according to the presence of chronic hypertension, defined as hypertension in antihypertensive therapy. Table S7. Arterial blood pressure according to the presence of subarachnoid hemorrage (SAH) on admission. Table S8. Arterial blood pressure according to patient sex. (DOCX 133 kb) [file 12871_2019_844_MOESM1_ESM.docx]

**Estimation of central arterial pressure from the radial artery in patients undergoing invasive neuroradiologic procedures**

**Scolletta et al**

**Supplemental Digital Content**

Additionnal file 1

**Table S1.** Individual values of central aortic pressure, peripheral pressure and aortic reconstructed pressure

| **Measure** | **SAP_ao_**  **mmHg** | **SAP_peripheral_**  **mmHg** | **SAP_rec_**  **mmHg** | **MAP_ao_**  **mmHg** | **MAP_peripheral_**  **mmHg** | **MAP_rec_**  **mmHg** | **DAP_ao_**  **mmHg** | **DAP_peripheral_**  **mmHg** | **DAP_rec_**  **mmHg** |
| --- | --- | --- | --- | --- | --- | --- | --- | --- | --- |
| **Patient 1** | 130.60 | 133.21 | 124.75 | 79.44 | 85.39 | 71.27 | 53.86 | 61.49 | 44.521 |
| **Patient 2** | 180.29 | 174.14 | 178.44 | 128.13 | 129.42 | 125.96 | 102.05 | 107.06 | 99.727 |
| **Patient 3** | 88.06 | 93.88 | 92.53 | 64.39 | 65.42 | 69.36 | 52.56 | 51.19 | 57.781 |
| **Patient 4** | 119.85 | 150.86 | 135.20 | 85.42 | 97.43 | 85.9 | 68.20 | 70.72 | 61.255 |
| **Patient 5** | 144.91 | 141.29 | 141.69 | 100.80 | 97.91 | 98.07 | 78.74 | 76.23 | 76.262 |
| **Patient 6** | 96.23 | 104.97 | 99.04 | 68.80 | 75.70 | 67.41 | 55.08 | 61.06 | 51.594 |
| **Patient 7.1** | 120.48 | 118.61 | 112.21 | 83.59 | 80.66 | 80.3 | 65.14 | 61.68 | 64.343 |
| **Patient 7.2** | 94.28 | 84.60 | 91.29 | 73.66 | 61.48 | 74.74 | 63.36 | 49.92 | 66.473 |
| **Patient 8** | 95.51 | 85.13 | 93.45 | 74.48 | 62.35 | 76.95 | 63.97 | 50.96 | 68.703 |
| **Patient 9** | 92.55 | 109.70 | 96.89 | 66.42 | 72.27 | 70.2 | 53.36 | 53.56 | 56.862 |
| **Patient 10** | 126.08 | 135.66 | 134.50 | 88.59 | 96.27 | 89.34 | 69.85 | 76.57 | 66.756 |
| **Patient 11** | 93.22 | 82.79 | 106.34 | 67.32 | 66.00 | 71.85 | 54.38 | 57.61 | 54.613 |
| **Patient 12** | 78.87 | 89.72 | 93.30 | 57.93 | 65.54 | 68.81 | 47.46 | 53.45 | 56.573 |
| **Patient 13** | 83.88 | 111.70 | 100.77 | 61.16 | 78.35 | 70.62 | 49.81 | 61.68 | 55.551 |
| **Patient 14.1** | 115.72 | 108.26 | 107.84 | 88.80 | 78.75 | 83.67 | 75.34 | 64.00 | 71.591 |
| **Patient 14.2** | 113.77 | 123.53 | 124.70 | 83.65 | 90.70 | 91.33 | 68.59 | 74.28 | 74.642 |
| **Patient 15.1** | 106.31 | 101.02 | 106.03 | 77.85 | 73.44 | 76.62 | 63.62 | 59.64 | 61.922 |
| **Patient 15.2** | 115.08 | 102.08 | 107.84 | 86.52 | 74.80 | 78.64 | 72.24 | 61.15 | 64.038 |
| **Patient 15.3** | 104.29 | 97.98 | 103.51 | 76.38 | 71.61 | 74.88 | 62.43 | 58.43 | 60.559 |
| **Patient 16** | 115.98 | 108.38 | 108.22 | 91.15 | 79.22 | 86.11 | 78.74 | 64.65 | 75.06 |
| **Patient 17** | 101.82 | 102.96 | 90.99 | 76.06 | 72.28 | 62.99 | 63.19 | 56.94 | 48.996 |
| **Patient 18** | 125.38 | 121.41 | 127.69 | 89.75 | 86.78 | 86.25 | 71.94 | 69.47 | 65.531 |
| **Patient 19** | 94.85 | 85.39 | 83.88 | 67.09 | 59.61 | 62.42 | 53.21 | 46.72 | 51.693 |
| **Patient 20** | 161.52 | 140.16 | 143.70 | 117.93 | 100.35 | 98.86 | 96.13 | 80.44 | 76.433 |
| **Patient 21** | 93.95 | 93.51 | 88.40 | 68.10 | 64.36 | 70.21 | 55.17 | 49.78 | 61.112 |

SAP_ao_, aortic systolic arterial pressure; SAP_peripheral_, peripheral systolic arterial pressure; SAP_rec_, reconstructed systolic pressure; DAP_ao_, aortic diastolic arterial pressure; DAP_peripheral_, peripheral diastolic arterial pressure; DAP_rec_, reconstructed diastolic pressure; MAP_ao_, mean aortic arterial pressure; MAP_peripheral_, peripheral mean arterial pressure MAP_rec_, reconstructed mean aortic arterial pressure.

**Figure S1.** Correlations between systolic (SAP_ao_), diastolic (DAP_ao_) and mean (MAP_ao_) arterial pressure recorded in the aorta and those recorded in the radial artery (SAP_peripheral_, DAP_peripheral_, MAP_peripheral_). Dotted lines represented the 95% confidence intervals


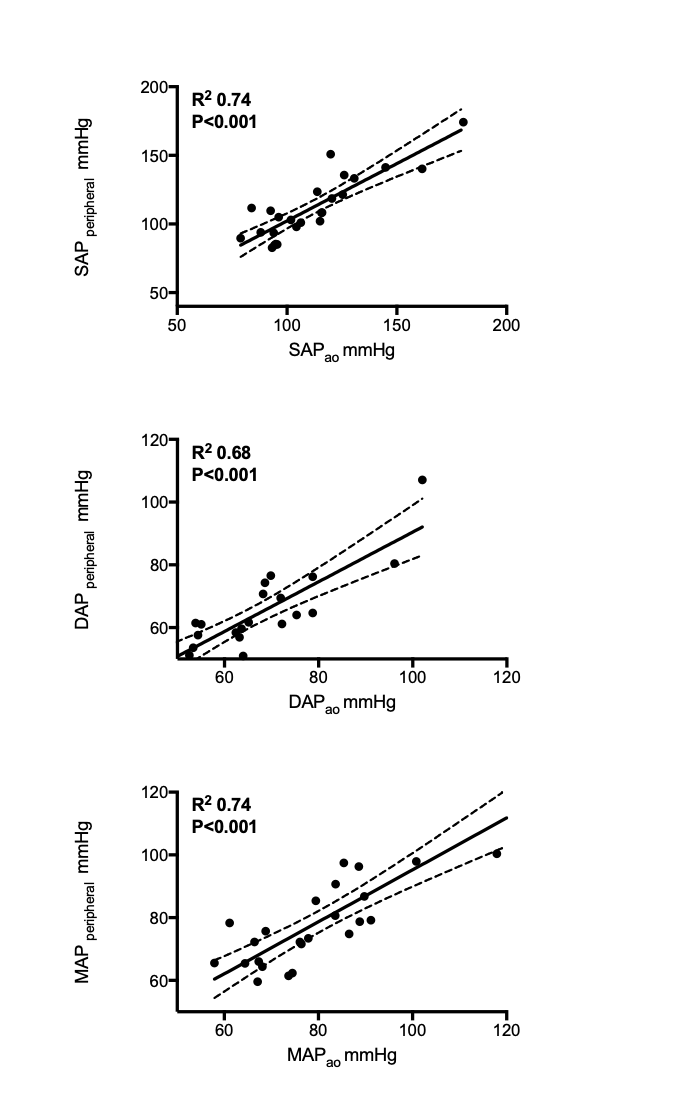


**Table S2.** Differences between arterial pressures recorded in the aorta and those recorded in the radial artery.

| **Variables** | **Bias** | **SE** | **95% CI** |
| --- | --- | --- | --- |
| SAP_ao_-SAP_peripheral_ mmHg | -0.3 | 2.5 | -5.4 to 4.5 |
| DAP_ao_-DAP_peripheral_ mmHg | 2.4 | 1.6 | -0.8 to 5.6 |
| MAP_ao_-MAP_peripheral_ mmHg | 1.5 | 1.7 | -2.1 to 5.1 |

SE, standard error; 95% CI, 95% confidence interval; SAP_ao_, aortic systolic pressure; SAP_peripheral_, peripheral systolic pressure; DAP_ao_, aortic diastolic pressure; DAP_peripheral_, peripheral diastolic pressure; MAP_ao_, aortic mean arterial pressure; MAP_peripheral_, peripheral mean arterial pressure.

**Table S3.** Linear regression of measured and reconstructed central arterial pressure values.

| **Variables** | **Equation** | **95% Confidence Interval** | | **R^2^** | **P** |
| --- | --- | --- | --- | --- | --- |
|  |  | **Slope** | **Intercept** |  |  |
| SAP_ao_ *vs* SAP_rec_ mmHg | Y=0.85X + 16.25 | 0.70 to 1.01 | -1.15 to 33.65 | 0.85 | <0.0001 |
| DAP_ao_ *vs* DAP_rec_ mmHg | Y=0.74X + 15.40 | 0.55 to 0.92 | 3.25 to 27.54 | 0.75 | <0.0001 |
| MAP_ao_ *vs* MAP_rec_ mmHg | Y=0.77X + 17.49 | 0.62 to 0.91 | 5.68 to 29.30 | 0.84 | <0.0001 |
| PP_ao_ *vs* PP_rec_ mmHg | Y=1.09X + -2.17 | 0.84 to 1.33 | -13.94 to 9.60 | 0.78 | <0.0001 |

SAP_ao_, aortic systolic arterial pressure; SAP_rec_, reconstructed systolic pressure; DAP_ao_, aortic diastolic arterial pressure; DAP_rec_, reconstructed diastolic pressure; MAP_ao_, mean aortic arterial pressure; MAP_rec_, reconstructed mean aortic arterial pressure; PP_ao_, aortic pulse pressure; PP_rec_, reconstructed aortic pulse pressure.

**Table S4.** Numbers of patients with >10 mmHg differences between central and reconstructed pressures

|  | **Number of patients with a difference greater than 10 mmHg between central and reconstructed pressure** | **Number of patients in whom reconstructed pressure underestimated actual central pressure** | **Comment** |
| --- | --- | --- | --- |
| **Systolic** | **8** | **3** | two of these patients also had a > 10 mmHg difference between  central and peripheral systolic pressure |
| **Diastolic** | **2** | **2** | one of these patients also had a > 10 mmHg difference between central and peripheral diastolic pressure |
| **Mean** | **3** | **2** | two of these patients also had a > 10 mmHg difference between reconstructed and measured systolic and diastolic pressure values; the remaining patient had a > 10 mmHg difference only between reconstructed and measured diastolic pressure |

**Table S5.** Arterial blood pressure according to norepinephrine therapy

|  | **No norepinephrine**  **(n=10)** | **Norepinephrine (n=15)** | **p** |
| --- | --- | --- | --- |
| SAP_ao_, mmHg | 105.5±14.1 | 115.9±28.6 | 0.927 |
| SAP_peripheral_, mmHg | 108.7±20.1 | 114.2±25.7 | 0.576 |
| SAP_rec_, mmHg | 108.6±15.0 | 113.8±26.3 | 0.576 |
| DAP_ao_, mmHg | 63.5±9.2 | 66.9±15.9 | 0.548 |
| DAP_peripheral_, mmHg | 61.8±7.3 | 64.0±15.8 | 0.691 |
| DAP_rec_, mmHg | 60.7±9.2 | 64.0±15.8 | 0.289 |
| MAP_ao_, mmHg | 77.5±10.5 | 83.2±19.6 | 0.408 |
| MAP_peripheral_, mmHg | 77.5±11.1 | 80.8±18.8 | 0.625 |
| MAP_rec_, mmHg | 76.6±8.4 | 81.8±16.5 | 0.375 |
| PP_ao_, mmHg | 41.9±7.0 | 49.0±16.0 | 0.147 |
| PP_peripheral_, mmHg | 46.9±14.5 | 50.2±12.2 | 0.541 |
| PP_rec_, mmHg | 4.9±13.2 | 48.0±18.8 | 0.984 |
| SAP_ao_- SAP_peripheral_, mmHg | -3.3±15.5 | 1.7±10.0 | 0.338 |
| SAP_ao_- SAP_rec_, mmHg | -3.1±10.1 | 2.1±8.2 | 0.168 |
| DAP_ao_- DAP_peripheral_, mmHg | 1.6±8.0 | 2.8±7.9 | 0.706 |
| DAP_ao_- DAP_rec_, mmHg | 2.8±6.0 | 1.2±7.2 | 0.555 |
| MAP_ao_- MAP_peripheral_, mmHg | 0.0±9.6 | 2.4±8.2 | 0.500 |
| MAP_ao_- MAP_rec_, mmHg | 0.9±6.5 | 1.4±7.0 | 0.833 |
| PP_ao_- PP_peripheral_, mmHg | -4.9±11.4 | -1.2±6.4 | 0.303 |
| PP_ao_- PP_rec_, mmHg | -6.0±8.2 | 0.9±6.3 | 0.024 |

SAP_ao_, aortic systolic arterial pressure; SAP_peripheral_, peripheral systolic arterial pressure; SAP_rec_, reconstructed systolic pressure; PP_ao_, aortic pulse pressure; PP_peripheral_, peripheral pulse pressure; PP_reconstructed_, reconstructed pulse pressure; DAP_ao_, aortic diastolic arterial pressure; DAP_peripheral_, peripheral diastolic arterial pressure; DAP_rec_, reconstructed diastolic pressure; MAP_ao_, mean aortic arterial pressure; MAP_peripheral_, peripheral mean arterial pressure MAP_rec_, reconstructed mean aortic arterial pressure.

**Table S6.** Arterial blood pressure according to the presence of chronic hypertension, defined as hypertension in antihypertensive therapy

|  | **No hypertension**  **(n=11)** | **Hypertension**  **(n=14)** | **p** |
| --- | --- | --- | --- |
| SAP_ao_, mmHg | 109.8±27.5 | 113.3±22.0 | 0.728 |
| SAP_peripheral_, mmHg | 113.3±30.2 | 111.2±17.4 | 0.842 |
| SAP_rec_, mmHg | 112.7±28.4 | 111.0±17.1 | 0.854 |
| DAP_ao_, mmHg | 64.8±14.4 | 66.1±13.2 | 0.827 |
| DAP_peripheral_, mmHg | 63.7±17.2 | 62.7±9.0 | 0.841 |
| DAP_rec_, mmHg | 63.3±13.8 | 64.0±9.8 | 0.879 |
| MAP_ao_, mmHg | 79.8±18.6 | 81.8±15.4 | 0.776 |
| MAP_peripheral_, mmHg | 80.2±21.1 | 78.8±11.4 | 0.837 |
| MAP_rec_, mmHg | 79.7±17.8 | 79.7±10.6 | 0.993 |
| PP_ao_, mmHg | 44.9±14.0 | 47.2±13.4 | 0.688 |
| PP_peripheral_, mmHg | 49.4±16.0 | 48.5±10.6 | 0.875 |
| PP_rec_, mmHg | 49.4±19.1 | 46.9±14.8 | 0.722 |
| SAP_ao_- SAP_peripheral_, mmHg | -3.3±15.7 | 2.1±9.1 | 0.290 |
| SAP_ao_- SAP_rec_, mmHg | -2.9±9.7 | 2.3±8.4 | 0.166 |
| DAP_ao_- DAP_peripheral_, mmHg | 1.1±8.1 | 3.4±7.7 | 0.475 |
| DAP_ao_- DAP_rec_, mmHg | 1.5±6.0 | 2.0±7.4 | 0.858 |
| MAP_ao_- MAP_peripheral_, mmHg | -0.4±9.5 | 2.9±8.0 | 0.352 |
| MAP_ao_- MAP_rec_, mmHg | 0.1±5.8 | 2.1±7.4 | 0.467 |
| PP_ao_- PP_peripheral_, mmHg | -4.4±12.4 | -1.3±4.1 | 0.391 |
| PP_ao_- PP_rec_, mmHg | -4.4±9.8 | 0.2±5.1 | 0.135 |

SAP_ao_, aortic systolic arterial pressure; SAP_peripheral_, peripheral systolic arterial pressure; SAP_rec_, reconstructed systolic pressure; PP_ao_, aortic pulse pressure; PP_peripheral_, peripheral pulse pressure; PP_reconstructed_, reconstructed pulse pressure; DAP_ao_, aortic diastolic arterial pressure; DAP_peripheral_, peripheral diastolic arterial pressure; DAP_rec_, reconstructed diastolic pressure; MAP_ao_, mean aortic arterial pressure; MAP_peripheral_, peripheral mean arterial pressure MAP_rec_, reconstructed mean aortic arterial pressure.

**Table S7.** Arterial blood pressure according to the presence of subarachnoid hemorrage (SAH) on admission

|  | **No SAH**  **(n=18)** | **SAH**  **(n=7)** | **p** |
| --- | --- | --- | --- |
| SAP_ao_, mmHg | 109.8±21.4 | 116.8±31.4 | 0.522 |
| SAP_peripheral_, mmHg | 112.1±19.5 | 112.0±33.2 | 0.995 |
| SAP_rec_, mmHg | 112.1±17.7 | 110.7±33.0 | 0.892 |
| DAP_ao_, mmHg | 65.2±12.7 | 66.4±16.4 | 0.846 |
| DAP_peripheral_, mmHg | 63.5±9.2 | 62.1±20.7 | 0.809 |
| DAP_rec_, mmHg | 63.8±18.5 | 63.5±18.5 | 0.953 |
| MAP_ao_, mmHg | 80.0±15.4 | 83.2±20.4 | 0.678 |
| MAP_peripheral_, mmHg | 79.7±12.2 | 78.7±24.4 | 0.896 |
| MAP_rec_, mmHg | 79.9±10.3 | 79.2±21.7 | 0.919 |
| PP_ao_, mmHg | 44.5±10.1 | 50.4±20.2 | 0.337 |
| PP_peripheral_, mmHg | 48.5±12.3 | 49.9±15.5 | 0.818 |
| PP_rec_, mmHg | 48.3±13.7 | 47.2±23.6 | 0.883 |
| SAP_ao_- SAP_peripheral_, mmHg | -2.3±13.9 | 4.8±5.4 | 0.206 |
| SAP_ao_- SAP_rec_, mmHg | -2.3±9.6 | 6.1±3.9 | 0.005 |
| DAP_ao_- DAP_peripheral_, mmHg | 1.6±7.8 | 4.3±8.2 | 0.459 |
| DAP_ao_- DAP_rec_, mmHg | 1.4±6.8 | 2.9±6.7 | 0.622 |
| MAP_ao_- MAP_peripheral_, mmHg | 0.3±9.2 | 4.4±6.7 | 0.295 |
| MAP_ao_- MAP_rec_, mmHg | 0.2±7.0 | 4.0±5.3 | 0.208 |
| PP_ao_- PP_peripheral_, mmHg | -4.0±9.4 | 0.5±6.2 | 0.256 |
| PP_ao_- PP_rec_, mmHg | -3.8±7.7 | 7.7±5.5 | 0.039 |

SAP_ao_, aortic systolic arterial pressure; SAP_peripheral_, peripheral systolic arterial pressure; SAP_rec_, reconstructed systolic pressure; PP_ao_, aortic pulse pressure; PP_peripheral_, peripheral pulse pressure; PP_reconstructed_, reconstructed pulse pressure; DAP_ao_, aortic diastolic arterial pressure; DAP_peripheral_, peripheral diastolic arterial pressure; DAP_rec_, reconstructed diastolic pressure; MAP_ao_, mean aortic arterial pressure; MAP_peripheral_, peripheral mean arterial pressure MAP_rec_, reconstructed mean aortic arterial pressure.

**Table S8.** Arterial blood pressure according to patient sex

|  | **Female**  **(n=16)** | **Male**  **(n=9)** | **p** |
| --- | --- | --- | --- |
| SAP_ao_, mmHg | 111.0±22.4 | 113.0±28.2 | 0.848 |
| SAP_peripheral_, mmHg | 108.0±20.6 | 119.1±27.4 | 0.262 |
| SAP_rec_, mmHg | 109.0±19.7 | 116.6±26.9 | 0.424 |
| DAP_ao_, mmHg | 64.0±12.1 | 68.2±16.1 | 0.474 |
| DAP_peripheral_, mmHg | 60.5±10.4 | 67.8±16.1 | 0.180 |
| DAP_rec_, mmHg | 62.1±8.5 | 66.5±15.6 | 0.457 |
| MAP_ao_, mmHg | 79.7±14.9 | 83.1±19.9 | 0.631 |
| MAP_peripheral_, mmHg | 76.3±13.6 | 84.9±19.2 | 0.204 |
| MAP_rec_, mmHg | 77.7±10.6 | 83.2±18.6 | 0.360 |
| PP_ao_, mmHg | 47.0±13.8 | 44.8±13.5 | 0.709 |
| PP_peripheral_, mmHg | 47.5±11.6 | 51.3±15.5 | 0.495 |
| PP_rec_, mmHg | 46.8±17.1 | 50.1±16.0 | 0.643 |
| SAP_ao_- SAP_peripheral_, mmHg | 3.0±8.9 | -6.1±16.0 | 0.143 |
| SAP_ao_- SAP_rec_, mmHg | 2.0±7.7 | -3.6±10.9 | 0.146 |
| DAP_ao_- DAP_peripheral_, mmHg | 3.5±7.4 | 0.3±8.5 | 0.338 |
| DAP_ao_- DAP_rec_, mmHg | 1.9±7.0 | 1.7±6.5 | 0.942 |
| MAP_ao_- MAP_peripheral_, mmHg | 3.4±7.7 | -1.8±9.7 | 0.158 |
| MAP_ao_- MAP_rec_, mmHg | 1.9±6.5 | -0.1±7.1 | 0.484 |
| PP_ao_- PP_peripheral_, mmHg | -0.5±3.8 | -6.5±13.3 | 0.224 |
| PP_ao_- PP_rec_, mmHg | 0.1±6.6 | -5.3±8.7 | 0.092 |

SAP_ao_, aortic systolic arterial pressure; SAP_peripheral_, peripheral systolic arterial pressure; SAP_rec_, reconstructed systolic pressure; PP_ao_, aortic pulse pressure; PP_peripheral_, peripheral pulse pressure; PP_reconstructed_, reconstructed pulse pressure; DAP_ao_, aortic diastolic arterial pressure; DAP_peripheral_, peripheral diastolic arterial pressure; DAP_rec_, reconstructed diastolic pressure; MAP_ao_, mean aortic arterial pressure; MAP_peripheral_, peripheral mean arterial pressure MAP_rec_, reconstructed mean aortic arterial pressure.
